# SARS-CoV Sampling from 3 Portals

**DOI:** 10.3201/eid1101.040645

**Published:** 2005-01

**Authors:** Tommy R. Tong

**Affiliations:** * Princess Margaret Hospital, Hong Kong, Special Administrative Region, China

**Keywords:** letter, SARS, SARS-CoV, saliva, tear, conjunctiva

**To the Editor:** Wang et al. detected severe acute respiratory syndrome–associated coronavirus (SARS-CoV) from throat wash and saliva specimens and suggested that these specimens have advantages over other specimens, including ease of procurement and safety for medical personnel (*1*). The virus has been detected with variable success from nasopharyngeal aspirates, nose and throat swabs, and tears (*2*,*3*). Advocates of all of these sampling methods emphasize the need for early diagnosis of SARS. The probability for nosocomial transmission to healthcare workers when they obtain specimens from patients has not been adequately addressed. In a study of outbreak control for SARS, Chowell et al. suggest “… the strong sensitivity of *R_0_* to the transmission rate β indicates that efforts in finding intervention strategies that manage to systematically lower the contact rate of persons of all age groups promise an effective means for lowering *R_0_*” (*4*).

An important component of a comprehensive strategy to lower the contact rate is improving the safety measures recommended for clinical specimen collection by healthcare workers. Recognizing the importance of obtaining multiple specimens and the difficulties associated with obtaining samples from the 3 usual portals of entry, we devised and tested a novel method of specimen collection, conjunctiva–upper respiratory tract irrigation (*5*). We coupled our specimen collection method with detailed written instructions to enable the patients themselves to perform the entire procedures. Almost all other specimen collection methods require assistance from healthcare workers or have other limitations, such as inability to sample all 3 portals. The method is not perfect because some persons have difficulty performing the procedure; however, self-instillation of the irrigation into the nostrils, with or without the addition of a throat wash or saliva, is likely to improve the success rate. The data supplied by Loon et al. (*3*) and Wang et al. (*1*) confirm that collecting specimens by a method that involves minimal contact between a possible source of infection and susceptible persons is desirable.
